# Comparisons of regular and on-demand regimen of PED5-Is in the treatment of ED after nerve-sparing radical prostatectomy for Prostate Cancer

**DOI:** 10.1038/srep32853

**Published:** 2016-09-09

**Authors:** Shi. Qiu, Zhuang Tang, Linghui Deng, Liangren Liu, Ping Han, Lu Yang, Qiang Wei

**Affiliations:** 1 Department of Urology, Institute of Urology, West China Hospital, West China Hospital, Sichuan University, Chengdu 610041, Sichuan, P. R. China; 2 Stroke Clinical Research Unit, Department of Neurology, West China Hospital, Sichuan University, Chengdu 610041, Sichuan, P. R. China

## Abstract

Phosphodiesterase type-5 inhibitors (PDE5-Is) have been recommended as first line therapy for erectile dysfunction for patients received nerve-sparing radical prostatectomy for prostate cancer. We examed the efficiency of PDE5-Is and considered the optimal application. Systematic search of PubMed, Embase and the Cochrane Library was performed to identify all the studies. We identified 103 studies including 3175 patients, of which 14 were recruited for systematic review. Compared with placebo, PDE5-Is significantly ameliorated the International Index of Erectile Function-Erectile Function domain score (IIEF) scores (MD 4.89, 95% CI 4.25–5.53, p < 0.001). By network meta-analysis, sildenafil seems to be the most efficiency with a slightly higher rate of treatment-emergent adverse events (TEATs), whereas tadalafil had the lowest TEATs. In terms of IIEF scores, regular regimen was remarkably better than on-demand (MD 3.28, 95% CI 1.67–4.89, *p* < 0.001). Regular use was not associated with higher proportion of patients suffering TEATs compared with on-demand (RR 1.02, 95% CI 0.90–1.16, p = 0.72). Compared with placebo, PDE5-Is manifested significantly improved treatment outcomes. Overall, regular regimen demonstrated statistically pronounced better potency than on-demand. Coupled with the comparable rate of side effects, these findings support the regular delivery procedure to be a cost-effective option for patients.

Prostate cancer (PCa) is the most common neoplasm diagnosed in men in the United States and Europe. One in six men is diagnosed with PCa and 94% of these patients are diagnosed with a clinically localized form of PCa, which can be treated via nerve-sparing radical prostatectomy (NSRP)^1^,^2^. Erectile dysfunction (ED) is the most common complication which has the potential to impact negatively on patients’ quality of life^3^. The pathophysiology of ED following RP (radical prostatectomy) primarily results from three causes: neural injury, vascular injury, and smooth muscle damage^4^. The cavernosal nerve and corresponding blood vessels are always disrupted, injured or transected during RP due to their proximity to the prostate and entire remove of the tumor. Phosphodiesterase type-5 inhibitors (PDE5-Is) prolongs cavernosal smooth muscle relaxation and facilitates erection by means of inhibiting the degradation of cyclic guanosine monophosphate in penile tissues^5^,^6^,^7^. Notwithstanding less effective than in the general population, phosphodiesterase type-5 inhibitors appear to be effective in the patients with post-NSRP ED^7^.

The influence and treatment strategy of PDE5-Is used to treat ED after NSRP have been largely investigated. However, previous records have failed to provide final evidence to promote the optimal regimen^8^,^9^,^10^. PDE5-Is had been administered on demand with a huge dosage conventionally. But dosing frequency should be a noteworthy consideration in developing effective approaches to ED. Then, regarding the effectiveness that lasts up to 36 hours, daily use of tadalafil allows patients a wide window to decide the time to engage in sexual intercourse^11^,^12^. Nevertheless, sufferers from ED preferred a cost-minimization and easy administration approach with comparable efficacy as well as treatment-emergent adverse events (TEAEs). Thus most recently, a new dosing regimen, three times/week was encouraged to the prescribing clinicians for its efficacy and well-tolerated property^13^. However, disparate method has both advantages and drawbacks, there is no consensus or guidelines on their application at present. In this case, PDE5-Is are often used more experientially without superior evidence-based medicine support. In this study, we are the first to combined regimen of daily use with three times/week and put forward a new thesis of “regular use”.

Thus, we conducted a systematic review to investigate the effectiveness and feasibility of PDE5-Is for ED following NSRP for PCa, and optimal way of drug delivery. Further studies on other influential factor will be summarized in our next work.

## Result

### Search Results

**Figure 1:**
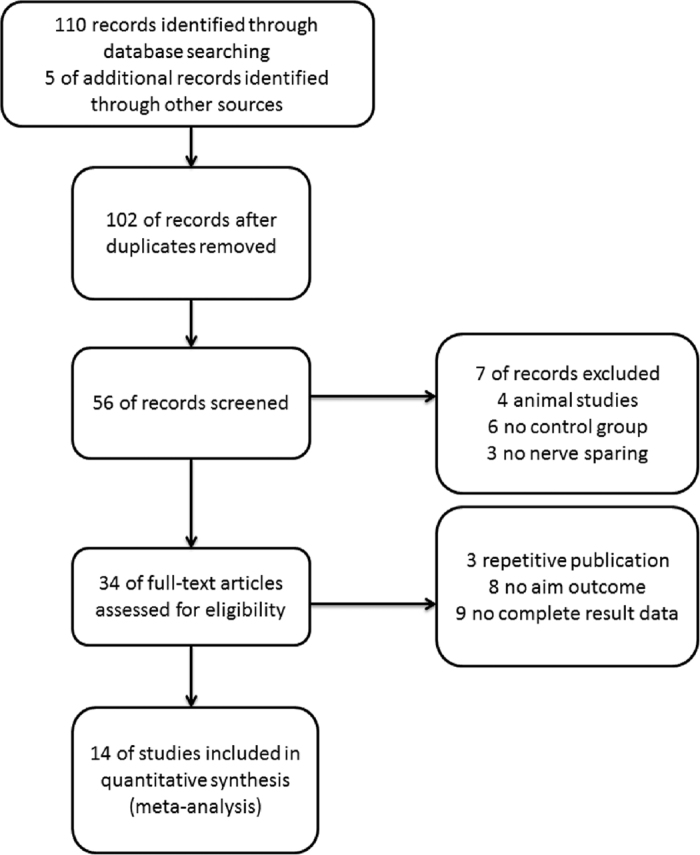
Flowchart of identification and selection of studies for the systematic review.

A total of 115 articles were identified through the electronic. A manual review of reference lists of involved studies complemented the search. (Figure 1) 34 articles were judged to be relevant considering the review of the titles and abstracts. More studies were eliminated for the use of inadequate study designs, and lack of relevance of measured outcome underlying full review. A total of 14 studies met all inclusion criteria and were involved in the pooled qualitative review. The characteristics of the studies are shown in Table 1.

### Characteristics of included studies

A total of 3,175 patients were recruited in the final meta-analysis, all of whom were diagnosed with ED after unilateral NS-RP (24%) or bilateral NS-RP (76%). The Mean patient age (years) ranged from 18 to 75 (median 24 to 77). Open surgery (53%), Conventional laparoscopy (34%), Robot-assisted laparoscopy (13%) were substantially used as NSRP modality. Follow-up ranged from 3 to 13 months. Included study populations were from the USA, UK, Italy, Netherlands, Spain, German, Turkey, Canada and Korea.

### Risk of bias

**Figure 2:**
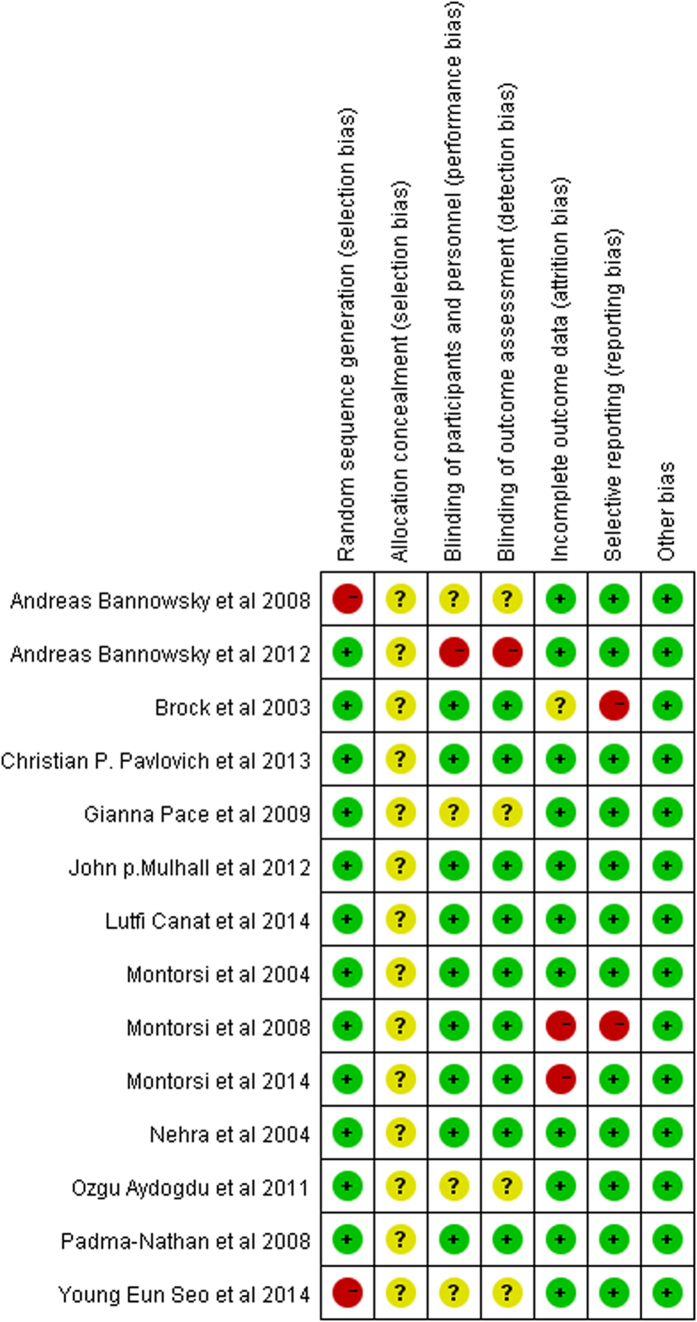
Risk of bias detail. Green circles represent positive risk of bias. Red circles indicate negative risk of bias. Yellow circles signify unknown risk of bias.

A total of 13 captured studies^14^,^15^,^16^,^17^,^18^,^19^,^20^,^21^,^22^,^23^,^24^,^25^ had adequate randomization. One study^26^ was randomized according to age, preoperative IIEF score and status of bilateral NS-RP. The major bias of involved studies was the lack of allocation concealment. None of the studies manifested method of allocation concealment and described their approach. Both researchers and patients were reported as blinded in 10 studies^15^,^16^,^19^,^20^,^21^,^22^,^23^,^24^,^25^. Secondly, only trial^25^ provided a placebo that was similar to the PDE5-Is, whereas the remainder offered no treatment in the control group. Accordingly, those studies were indicated to have high potential for performance biases. Furthermore, five studies did not describe their blindness method^17^,^18^,^26^,^27^ and one study^14^ was not blinded. This can be contributed to the prevalent of detection bias. The selective reporting was considered as ‘high risk of bias’ in two studies^6^,^21^. The overall quality of the included studies is high. There was little visual evidence of publication bias in funnel plots (Supplementary Fig. S1). Figure 2 illustrated the authors’ judgments on each of the risk of bias domain for each research.

Firstly, we performed a meta-analysis of the studies reporting the result of PDE5-Is treating ED: 14 studies of 3,175 patients^14^,^15^,^16^,^17^,^18^,^19^,^20^,^21^,^22^,^23^,^24^,^25^,^26^,^27^ according to IIEF scores and treatment-emergent adverse events (TEAEs).

**Figure 3:**
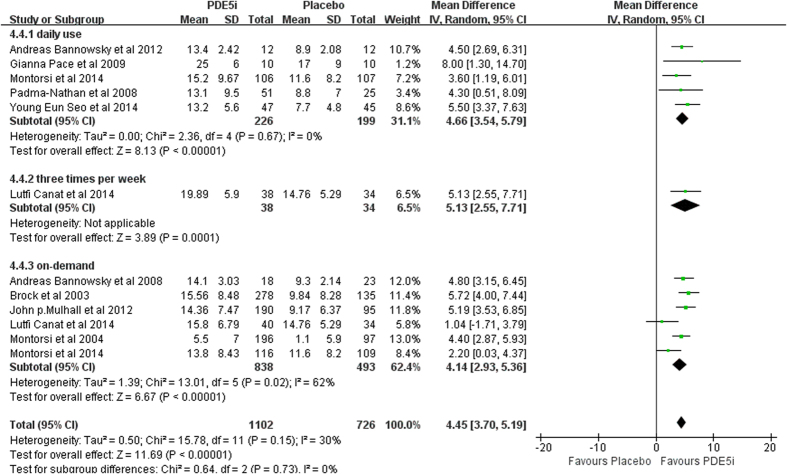
Forest plot of PDE5-Is efficacy versus placebo: Mean difference (MD) of IIEF score. Squares indicate odds ratios. Diamonds represent summary measures (center of diamond) and associated confidence intervals (lateral tips of diamond).

IIEF score. Patients who had undergone PDE5-Is procedure exhibited a significant merit to the placebo group on the types of PDE5-Is (fixed effects model: MD 4.89, 95% CI 4.25–5.53, *p* < 0.001). In the subgroup synthesis, the efficacy of regular use (daily use and 3 times/week) was comparable with on-demand (fixed effects model: MD 4.66, 95% CI 3.54–5.79, *p* < 0.001; MD 5.13, 95% CI 2.55–7.71; MD 4.99, 95% CI 4.17–5.81, *p* < 0.001). (Figure 3).

**Figure 4:**
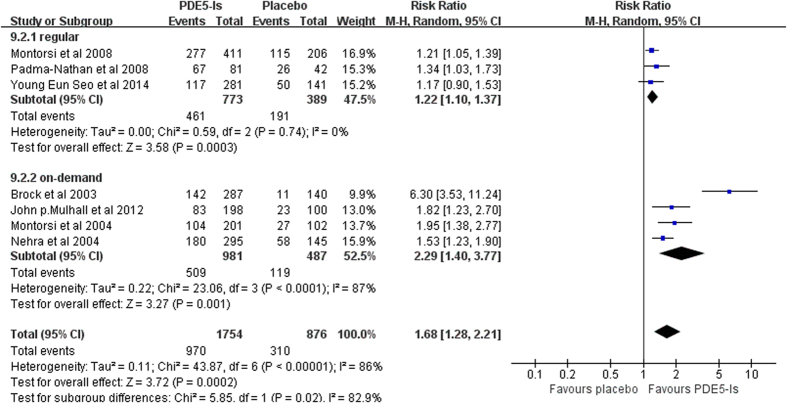
Forest plot of PDE5-Is safety versus placebo: Relative risk (RR) of TEAEs rate.

TEAEs. When comparing patients suffering TEAEs with placebo group, significant differences were found in both regular and on-demand use (random effects model: RR 1.22, 95% CI 1.10–1.37, *p* = 0.0003; RR 2.29, 95% CI 1.40–3.77, *p* = 0.001). Additionally, a tendency towards higher TEAEs incidence was visualized in on-demand group. (Figure 4) Sensitivity analysis was conducted by excluding each of the studies and the pooled results remained statistically significant.

**Figure 5:**
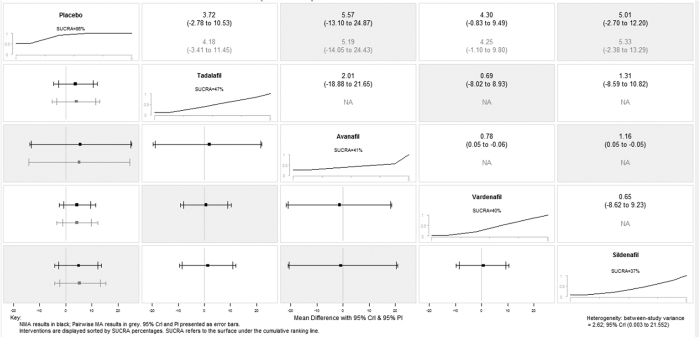
Summary forest plot matrix for placebo versus PDE5-Is efficacy. NMA, network meta-analysis; CrI, credible interval; PI, prediction interval

**Figure 6:**
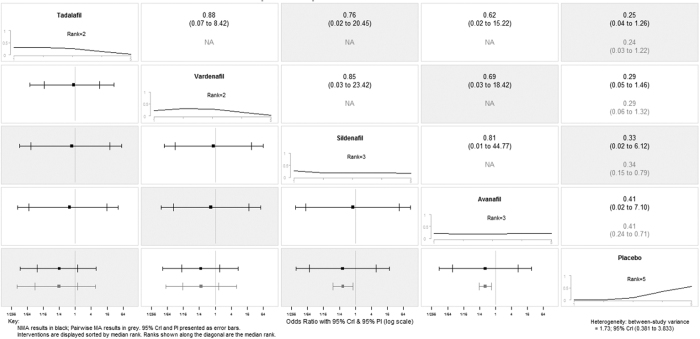
Summary forest plot matrix for PDE5-Is safety versus placebo. NMA, network meta-analysis; CrI, credible interval; PI, prediction interval.

We attempted to identify the potential sources of heterogeneity (I^2^ = 86%) through subgroup analysis based on disparate drugs on TEATs. The subgroup differences reduce remarkably from 82.9% to 4%, which means drug type could explain the source of heterogeneity. After strategizing for different drugs, the network meta-analysis estimations indicated that a majority of treatments were better than placebo. Ranking on efficacy suggested that Sildenafil treatment was the highest, followed by vardenafil, avanafil and tadalafil. However, the result should be interpreted with caution due to none of the comparisons reached statistical significance. (Figure 5 and supplementary Fig. S2). Confidence intervals were wide in pairwise meta-analysis comparisons and credible intervals were not narrow in network meta-analysis comparisons, indicating the small number of trials available. On the number of patients presenting with TEAEs, the network meta-analysis revealed that tadalafil and vardenafil might be relatively optimal choices. Again, only two pairwise comparisons reached statistical significance. (Figure 6 and Supplementary Fig. S3). Data for pairwise comparisons and network estimates are shown. We ranked the comparative effects of all drugs against placebo with surface under the cumulative ranking (SUCRA) probabilities. Heterogeneity from between-study variance was generally small. Similarly, no statistically salient inconsistency was denoted in most loops within the network.

Secondly, we performed a meta-analysis of the four studies, 834 patients assessing the efficiency of different method of PDE5-Is.

**Figure 7:**
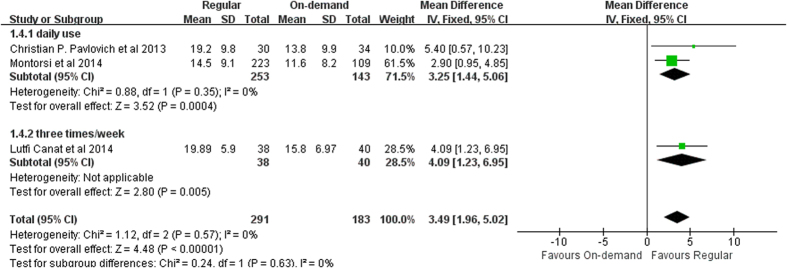
Forest plot of regular regimen efficacy versus on-demand group: Mean difference (MD) of IIEF score.

IIEF score before washout. With follow-up ranging from 9 to 13 months, on-demand group demonstrated statistically remarkable differences to the regular group (fixed effects model: MD 3.28, 95% CI 1.67-4.89, *p* < 0.001). Significant differences favoring 3 times/week treatment were identified compared with on-demand (MD 4.09, 95% CI 1.23–6.95, *p* = 0.005, I^2^ = 0). (Figure 7).

**Figure 8:**
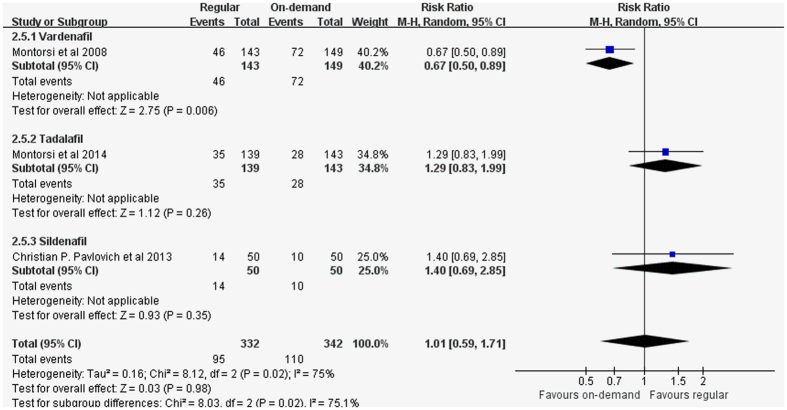
Forest plot of regular regimen efficacy versus on-demand group: Proportion of patients achieving IIEF score ≥22.

Proportion of patients achieving IIEF score ≥22. Pooled analysis revealed that when compared to the on-demand group, no significantly higher proportion of patients in regular use group reported achieving IIEF score ≥22 (random-effect model: RR 1.01, 95% CI 0.59-1.71, *p* = 0.02, I^2^ = 75%). (Figure 8) A retrospective research from Natali *et al*.^28^ also witness no statistically profound differences between on-demand and regular protocols (72% with regular and 70% with on-demand therapy). Meta-regression models based on year of publication, treatment duration, age at the trial level. From these variables, none was pronounced (*p* = 0.288, *p* = 0.64, *p* = 0.834, separately). Sensitivity analyses revealed no remarkable advance in statistical power after excluding each of the studies.

TEATs. Regular use was not associated with higher proportion of patients suffering side effects when compared to the on-demand (fixed-effect model: RR 1.02, 95% CI 0.90–1.16, *p* = 0.72). Stratified by the disparate dosing method, no saliently differences were found between either regimen (daily, three times/week) and on-demand use (RR 0.99, 95% CI 0.87–1.12, *p* = 0.88; RR 1.68, 95% CI 0.88–3.24, *p* = 0.12). (Supplementary Fig. S4)

## Discussion

The influence of PDE5-Is used to treat ED following BNSRP has been largely investigated. Although different efficacy has been reported, PDE5-Is dosing method for penile rehabilitation remains ill-defined. We are the first to put forward the thesis of regular use (daily and three times/week) and predominantly focused on the comparing of efficacy, feasibility and preference to the on-demand. Furthermore, we conducted a network meta-analysis to identify the optimal drugs. The present meta-analysis showed that PDE5-Is were efficacious among the specific ED patients. Regular use significantly ameliorated ED following BNSRP compared with on-demand. One of the main findings of our meta-analysis seems to contradict the previous meta-analysis^29^. They revealed that the on-demand use might be more effective than daily administration in improving IIEF score. Their findings could be better convinced if they demonstrated statistical differences.

Both the previous meta-analysis^29^,^30^,^31^ and ours revealed that a larger portion of ED patients considerably benefited from PDE5-Is intervention when compared with placebo. After stratified by various drugs, we found that all four drug regimen ameliorated IIEF score compared with placebo both in the network and pairwise meta-analysis. Sildenafil procedure seems to be the most efficiency with a slightly higher rate of TEATs, followed by vardenafil, avanafil and tadalafil, whereas tadalafil had the lowest TEATs. Besides, sensitivity analyses based on various exclusion criteria did not alter the pooled effect, which strengthening our result. Regarding dosing method, the current result suggested statistically salient differences between the regular dosing group and the on-demand group. Regarding of three times per week, we witness a better performance than daily or nightly use.

Inextricably associating the medication with a man’s sexual performance, PDE5-Is for ED have empirically been used just before a sexual encounter^32^. Pharmacokinetic studies of tadalafil confirmed that the steady state is reached after five day of daily use^30^,^32^. A total tadalafil plasma concentration of 55 ng/ml constituted a reasonable pharmacodynamic target, suggesting the maintenance of these concentrations throughout the dosing interval of 24 h^33^. In the trial of Porst *et al*., tadalafil remarkably increase the percentage of successful intercourse attempts. It was still evident 36 hours after dosing^34^. Overall, regular use, especially 3 times/week regimen demonstrated better potency of ED after NSPR than on-demand use.

In light of TEATs, regular use and on-demand use both associated with high TEAEs occurrence, but regular group manifested lower rate. Headache was the most common side effect (15.8% vs. 10%) along with flushing (15.8% vs. 10%)^15^. Notwithstanding the tadalafil plasma concentration would be expected to change on the basis of the dosing method, this synthesis confirmed that the incidence of serious TEAEs were comparable among men with erectile dysfunction who took tadalafil as needed, three times/week, or daily^15^. After all, coupled with the low rate of TEAEs, these findings seem to support regular dosing method to be reasonable.

In terms of patients’ preference, in the trial of Mirone *et al*. a large number of patients (42.2% vs 57.8%, P = 0.381) preferred the three times/week method^11^. We may indicate a pronounced percentage of patients consider this method efficacious and desirable. Tadalafil has been demonstrated to be efficacious up to 36 hours after using for a half-life of 17.5 hours^34^. In particular, a three times/week regimen provides nearly continuous coverage. Thus, patients with ED can choose when to have a sexual encounter with greater flexibility^11^. For instance, tadalafil enables a patient to take a pill on a Friday morning and have intercourse with his partner on Saturday morning or night. Moreover, regarding to the comparable efficacy, taking PDE5-Is three times/week may be more cost-effective than daily use. A two times/week dosing or less can’t provide a broad therapeutic coverage, thus might translate to compromised efficacy and unnecessarily complicate administration. Following five days of the daily use of tadalafil, steady-state plasma concentrations are reached which provides a 1.6 times greater exposure than single oral dose or three times/week method^35^. This would result in increased accidence of adverse events as well. Furthermore, considering the comparable efficacy, taking PDE5-Is three times/week may be more cost-effective than four times/week. In this regard, three times/week regimen could be recommended regarding to the quality of life for patients compared with four times/week or more frequency.

We must acknowledge that, in general, meta-analyses carry limitations. The patients TEATs rate and proportion of patients achieving IIEF score ≥22 had high heterogeneity (I^2^ = 86% and 75%). Factors that could conceivably explain the heterogeneity involve various trial design schemes, surgeries performed by surgeons with varying experience, diverse drug type and patients baseline oncological characteristics. An individual participant meta-analysis might overcome this flaw even if this might reduce the sample size. Additionally, the primary outcomes are all assessed by patient reported outcome apart from laboratory data. Further clinical research based on objective measurement is desired. Fewer dosing is associated with improved compliance, which in turn may link up with lower healthcare resource use and cost. However, we did not emphasize specifically on the relationship between healthcare resource utilization and various dosing method. Further research is needed to inform on this issue.

## Methods

### Search Strategy

We followed the ‘Preferred Reporting Items for Systematic Reviews and Meta-Analyses’ (PRISMA) guidelines. We searched the underlying databases: PubMed (from 1998 to January 2016), Embase (from 1998 to January 2016) and the Cochrane Database of Systematic Reviews. An update systematic search was done in June 2016. Reference lists of included studies were manually screened for any additional series. The initial search was designed to identify all trials involving the underlying keywords in combination with medical subject headings terms: phosphodiesterase type 5 inhibitor or sildenafil or tadalafil or avanafil or vardenafil plus radical prostatectomy plus erectile dysfunction. Articles were limited to humans, gender (male).

### Selection Criteria

Inclusion criteria consisted of heterosexual adult male patients who had ED following BNSRP. We design to include only RCTs. Specific studies including patients with particular conditions were excluded from the synthesis, particularly patients with previous pelvic surgery, current or prior chemotherapy treatment, or metastases. Additionally, patients with serious cardiovascular disease, patients using nitrates and patients with a history of spinal cord injury or stroke within six months before study entry were also excluded. There was no limitation on publication status or language. S.Q. and Z.T. independently reviewed the titles and abstracts to identify the relevant papers. Full-text articles were then evaluated against inclusion and exclusion criteria. Disagreements were disentangled in consultation with the third reviewer (Q.W.) until consensus was reached.

### Data Extraction

Two reviewers performed data extraction independently (Q.S. and H.P.). Data extraction included patient-level factors (age, IIEF score, treatment period, PDE5-Is regimen, PDE5-Is types, TEATs) and study level factors (year of publication, country of origin, study funding, conflict of interest disclosure). Disagreements were resolved via discussion. Population information and study characteristics were extracted independently using standard data extraction forms.

### Outcome Measures

Outcomes or complications were measured, including the IIEF questionnaire. The IIEF questionnaire, developed by Rosen *et al*. is an international, validated 15-item self-administered questionnaire that attempts to quantify aspects of sexual function (Table 2)^30^. The adverse effects evaluated were flushing, headache, dyspepsia and upper respiratory track complaints.

### Evidence Quality Assessment

The quality of the recruited studies was evaluated using the Cochrane risk of bias tool^13^. In particular, the underlying factors were considered: (1) Adequate sequence generation (2) Allocation concealment (3) Binding (4) Incomplete outcome data addressed (5) Free of selective reporting (6) Free of other bias. Every question was answered with “yes”, “no” or “unclear”. Two reviewers evaluated each trial. Disagreement between reviewers was addressed by discussion with a third reviewer (Q.W).

### Statistical Analysis

Traditional pairwise meta-analyses were carried out using RevMan 5.3^36^. Continuous outcomes were presented as MD, and dichotomous data were presented as RR, both with a 95% CI. The meta-analysis was employed with fixed effects method or random effects method. Heterogeneity among studies was valued with the Chi^2^ test and the Higgins-Thompson I^2^ method. The pronounced heterogeneity was indicated by a p-value < 0.05 and an I^2^ >50%, in which the randomised-effects models were executed. When substantial heterogeneity was not observed, we carried out fixed-effects models to estimate the pooled result. We did random-effects network meta- analysis within a Bayesian framework, using WinBUGS (version 1.4.3)^37^ and R software (version 2.13.0)^38^. To rank the procedure for an outcome, we used SUCRA probabilities, which express as a percentage the efficacy or safety of every intervention that is always the best without uncertainty. Publication bias was evaluated by funnel plot. We carried out meta-regression to assess the association between trial characteristics and pooled effects with metareg command in Stata^39^. Additionally, sensitivity analysis was performed if there were low-quality trials included in the analysis.

## Conclusion

In summary, this systematic review suggested that PDE5-Is were safe and efficacious in the treatment of ED after NSRP. Using network meta-analysis, sildenafil seems to be the most efficiency with a slightly higher rate of TEATs, followed by vardenafil, avanafil and tadalafil, whereas tadalafil had the lowest TEATs. Direct comparisons between regular and on-demand delivery of PDE5-Is demonstrated statistically significant difference. Given its better outcomes and decreased incidence of TEATs, regular use of tadalafil seems to be a reasonable management option in the treatment of ED after NSRP in PCa patients who wished to regain sexual function^40^,^41^,^42^.

## Additional Information

**How to cite this article**: Qiu, S. *et al*. Comparisons of regular and on-demand regimen of PED5-Is in the treatment of ED after nerve-sparing radical prostatectomy for Prostate Cancer. *Sci. Rep.* **6**, 32853; doi: 10.1038/srep32853 (2016).

## Supplementary Material

Supplementary Information

## Figures and Tables

**Table 1:** Characteristics of the included studies.

Study ID	Country	Sample size	Intervention(regular)	Intervention(on-demand)	Control	Treatment period	Outcome
Bannowsky^14^	German	24	vardenafil 5 mg/day	no treatment	no use of PDE5-Is	12 months	IIEF score
Canat^15^	Turkey	112	tadalafil 20 mg 3 times/week	tadalafil on demand	no use of PDE5-Is	12 months	IIEF score/ TEAEs
Montorsi^16^	Europe, USA, Canada and South Africa	628	vardenafil 10 mg/night plus on-demand placebo	vardenafil 10 mg on demand plus nightly placebo	nightly placebo plus on-demand placebo	9 months	IIEF score/Proportion of patients achieving IIEF score ≥22/ TEAEs
Aydogdu^17^	Turkey	85	tadalafil 20 mg/day	no treatment	no use of PDE5-Is	6 months	IIEF score
Bannowsky^26^	USA	43	sildenafil 25 mg/day	no treatment	no use of PDE5-Is	13 months	IIEF score
Pace^18^	Italy	62	sildenafil 50 or 100 mg/day	no treatment	no use of PDE5-Is	6 months	IIEF score/ TEAEs
Mulhall^19^	USA	298	No treatment	avanafil 100 mg or 200 mg on-demand	placebo on-demand	3 months	TEAEs
Nathan^20^	North America and France	125	sildenafil 50 mg/night	no treatment	placebo nightly	9 months	IIEF score/ TEAEs
Brock^21^	USA and Canada	440	vardenafil 10 mg/day	vardenafil 20 mg on-demand	placebo on-demand	3 months	IIEF score/ TEAEs
Montorsi^22^	Canada, Germany, Italy, Netherlands, Spain, USA and UK	303	No treatment	tadalafil 20 mg on-demand	placebo on-demand	3 months	IIEF score/ TEAEs
Montorsi^23^	Nine European countries and Canada	423	Tadalafil 5 mg/day	Tadalafil 20 mg on-demand	placebo on-demand	9 months	IIEF score/ Proportion of patients achieving IIEF score ≥22/ TEAEs
Nehra^24^	USA and Canada	440	No treatment	10 mg vardenafil or 20 mg vardenafil on-demand	placebo on-demand	3 months	IIEF score/ TEAEs
Young^27^	Korea	92	tadalafil 5 mg/day(47)	no treatment	no use of PDE5-Is	12 months	IIEF score/ TEAEs
Pavlovich^25^	USA	100	Sildenafil 50 mg/day and on-demand placebo	Sildenafil 50 mg on-demand and nightly placebo	no use of PDE5-Is	12 months	IIEF score/ Proportion of patients achieving IIEF score ≥22/TEAEs

Phosphodiesterase type-5 inhibitors (PDE5-Is); International Index of Erectile Function (IIEF);Treatment-emergent adverse events (TEAEs).

**Table 2:** International Index of Erectile Function (IIEF) questionnaire.

1. How often were you able to get an erection during sexual activity? (erection frequency)
2. When you had erections with sexual stimulation, how often were your erections hard enough for penetration? (erection firmness)
3. When you attempted sexual intercourse, how often were you able to penetrate (enter) your partner? (penetration ability)
4. During sexual intercourse, how often were you able to maintain your erection after you had penetrated (entered) your partner? (maintenance frequency)
5. During sexual intercourse, how difficult was it to maintain your erection to completion of intercourse? (maintenance ability)
6. How many times have you attempted sexual intercourse? (intercourse frequency)
7. When you attempted sexual intercourse, how often was it satisfactory to you? (intercourse satisfaction)
8. How much have you enjoyed sexual intercourse? (intercourse enjoyment)
9. When you had sexual stimulation or intercourse, how often did you ejaculate? (ejaculation frequency)
10. When you had sexual stimulation or intercourse, how often did you have the feeling of orgasm or climax? (orgasm frequency)
11. How often have you felt sexual desire? (desire frequency)
12. How would you rate your sexual desire? (desire level)
13. How satisfied have you been with your overall sex life? (overall satisfaction)
14. How satisfied have you been with your sexual relationship with your partner? (relationship satisfaction)
15. How do you rate your confidence that you could get and keep an erection? (erection confidence)

The IIEF questionnaire can be grouped into five domains: erectile function, questions 1–5 and 15; intercourse satisfaction, questions 6–8; orgasmic function, questions 9 and 10; sexual desire, questions 11 and 12; and overall satisfaction, questions 13 and 14. The responses were graded on a scale of 1 (almost never or never) to 5 (almost always or always), with 0 indicating no sexual activity.
