# Supplementary material for: Comparisons of regular and on-demand regimen of PED5-Is in the treatment of ED after nerve-sparing radical prostatectomy for Prostate Cancer
Source: Sci Rep. 2016 Sep 9;6:32853. doi: 10.1038/srep32853 (PMC5017160; doi:10.1038/srep32853)
Supplement: Supplementary Information [file srep32853-s1.pdf]

Comparisons of regular and on-demand regimen of PED5-Is in the treatment of ED after nerve-sparing radical prostatectomy for Prostate Cancer

Shi.Qiu[1#], Zhuang Tang[1#], Linghui Deng[2], Liangren Liu[1], Ping Han[1], Lu Yang[1]*, Qiang Wei[1]*

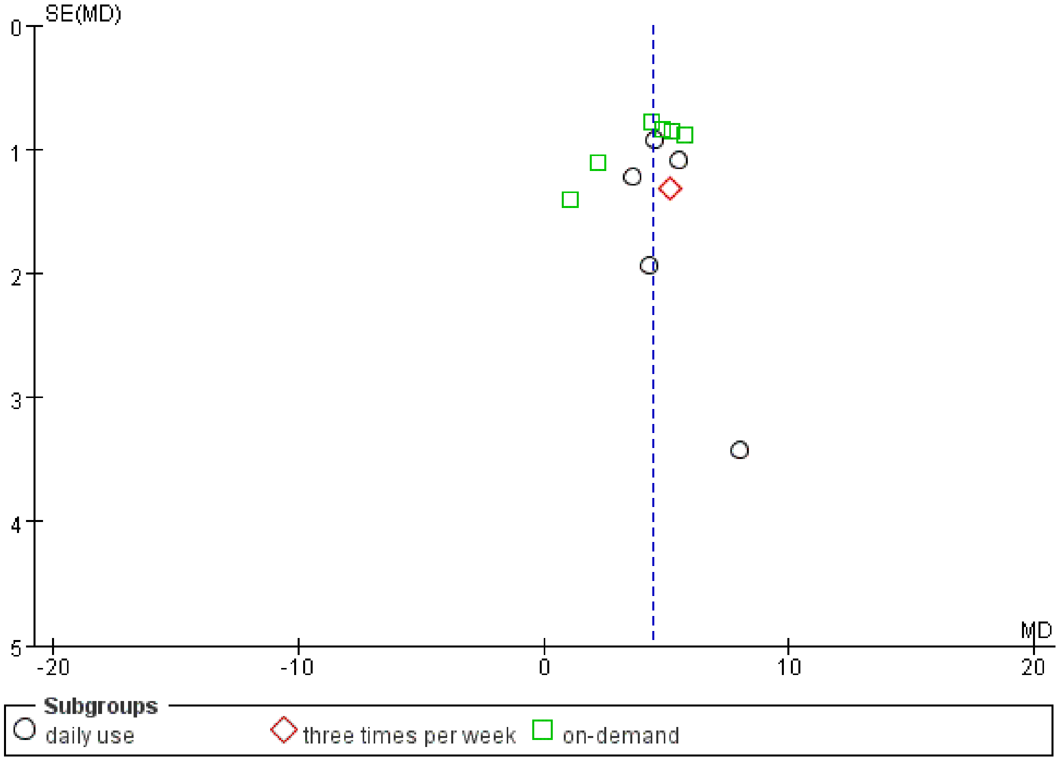

Supplementary figure S1. Funnel plot of included studies for placebo versus PDE5-Is efficacy. Circles indicate series with low risk of bias. Squares represent studies with higher risk of bias.

Comparators		SUCRA	H-H Trials	Mean Difference (95% CrI)
Placebo<br>SUCRA =86%	Tadalafil	SUCRA = 47%	6	3.72 (-2.78 to 10.53)<br>4.18 (-3.41 to 11.45)
	Avanafil	= 41%	1	5.57 (-13.10 to 24.87)<br>5.19 (-14.05 to 24.43)
	Vardenafil	= 40%	3	4.30 (-0.83 to 9.49)<br>4.25 (-1.10 to 9.80)
	Sildenafil	= 37%	3	5.01 (-2.70 to 12.20)<br>5.33 (-2.38 to 13.29)
Tadalafil	Avanafil		0	2.01 (-18.88 to 21.65)<br>NA
	Vardenafil		0	0.69 (-8.02 to 8.93)<br>NA
	Sildenafil		0	1.31 (-8.59 to 10.82)<br>NA
Avanafil	Vardenafil		0	0.78 (0.05 to -0.06)<br>NA
	Sildenafil		0	1.16 (0.05 to -0.05)<br>NA
Vardenafil	Sildenafil		0	0.65 (-8.62 to 9.23)<br>NA

Summary Forest Plot

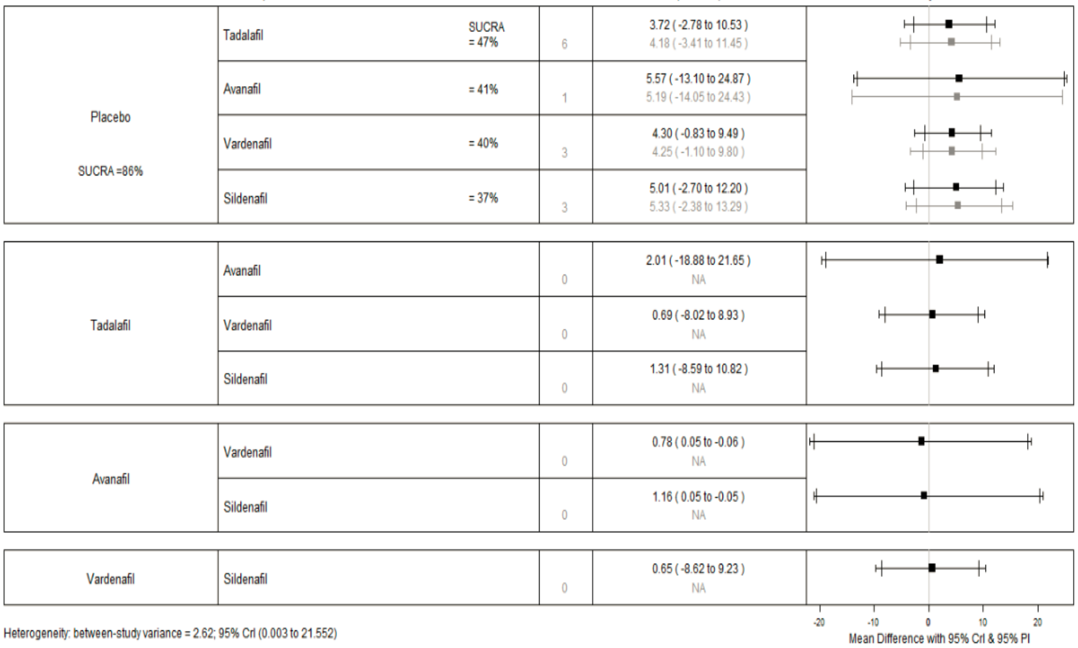

Heterogeneity: between-study variance = 2.62; 95% CrI (0.003 to 21.552)

Key: NMA results in black; Pairwise MA results in grey. 95% CrI and PI presented as error bars.
Interventions are displayed sorted by SUCRA percentages. SUCRA refers to the surface under the cumulative ranking line.

Supplementary figure S2. Summary forest plot table. H-H trials, head-to-head trials; NMA, network meta-analysis; CrI, credible interval; PI, prediction interval

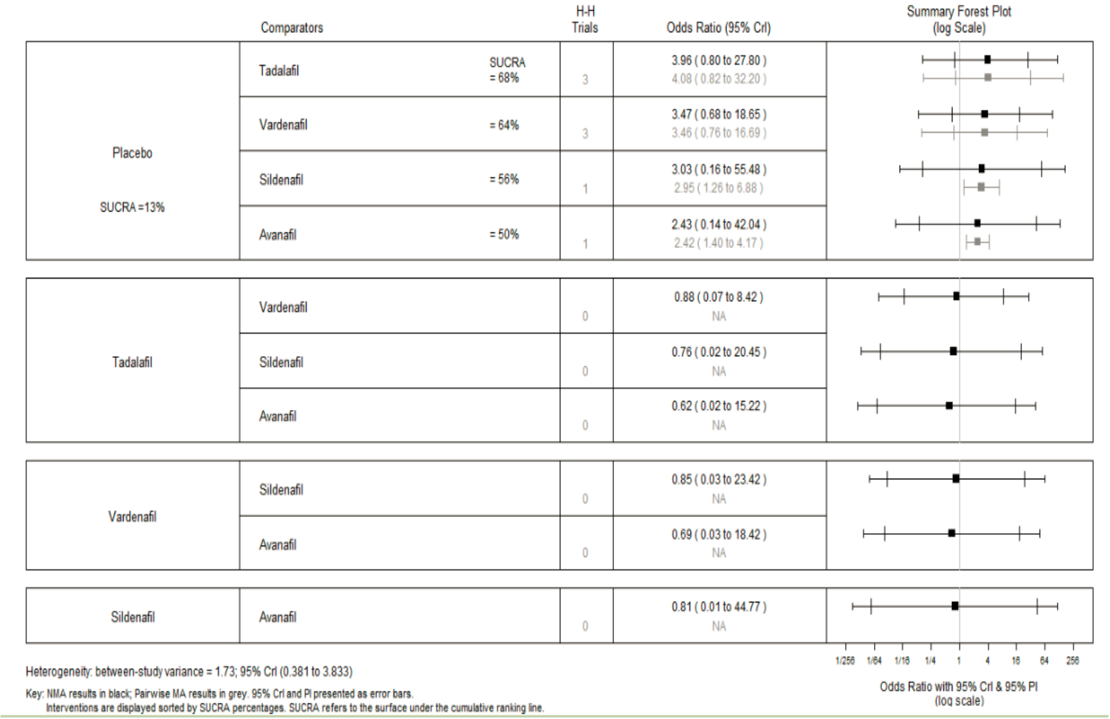

Supplementary figure S3. Summary forest plot table for PDE5-Is safety versus placebo. H-H trials, head-to-head trials; NMA, network meta-analysis; CrI, credible interval; PI, prediction interval

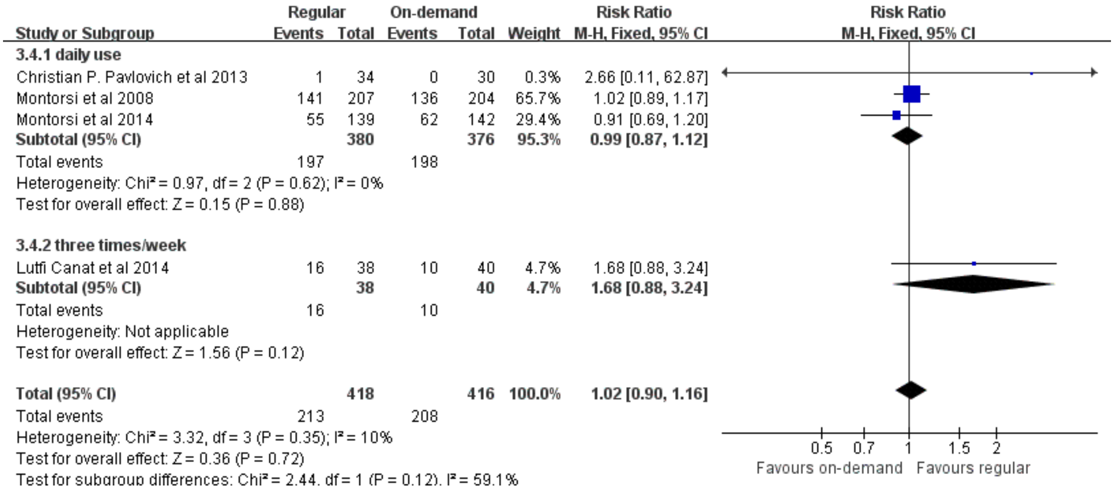

Supplementary figure S4. Forest plot of regular regimen safety versus on-demand group: Relative risk (RR) of TEAEs rate
